# Reflexology versus Swedish Massage to Reduce Physiologic Stress and Pain and Improve Mood in Nursing Home Residents with Cancer: A Pilot Trial

**DOI:** 10.1155/2012/456897

**Published:** 2012-07-24

**Authors:** Nancy A. Hodgson, Doreen Lafferty

**Affiliations:** ^1^Department of Acute and Chronic Care, School of Nursing, Johns Hopkins University, 525 N. Wolfe Street, Baltimore, MD 21205, USA; ^2^Genesis Rehabilitation Services, Kennett Square, PA 19348, USA

## Abstract

*Objective*. The purpose of this pilot study was to investigate and compare the effects of reflexology and Swedish massage therapy on physiologic stress, pain, and mood in older cancer survivors residing in nursing homes. *Methods*. An experimental, repeated-measures, crossover design study of 18 nursing home residents aged 75 or over and diagnosed with solid tumor in the past 5 years and following completion of cancer treatments. The intervention tested was 20 minutes of Swedish Massage Therapy to the lower extremities, versus 20 minute Reflexology, using highly specified protocols. Pre- and post-intervention levels of salivary cortisol, observed affect, and pain were compared in the Swedish Massage Therapy and Reflexology conditions. *Results*. Both Reflexology and Swedish Massage resulted in significant declines in salivary cortisol and pain and improvements in mood. *Conclusions*. Preliminary data suggest that studies of Swedish Massage Therapy and Reflexology are feasible in this population of cancer survivors typically excluded from trials. Both interventions were well tolerated and produced measurable improvements in outcomes. Further research is needed to explore the mechanisms underlying the potential benefits of these CAM modalities in this patient population.

## 1. Introduction

Cancer is a leading cause of morbidity and mortality in the older population. Demographic trends in the aging of the population, coupled with trends in cancer diagnoses and treatment, will shift much of the care of older cancer survivors to the nursing homes setting. Older cancer survivors suffer many long-term side effects of cancer and its treatment that threatens their quality of life [1]. Pain and distressing symptoms are common and often difficult to treat pharmacologically. Thus, investigations into the care of nursing home cancer survivors are particularly relevant.

Complementary therapy interventions have shown great promise in reducing distress and promoting comfort in cancer survivors [2, 3]. Two of the most widely accepted manual CAM therapies are reflexology and massage therapy. Recent reviews suggest that these modalities may have beneficial effects such as decreasing pain and increasing qualify of life in patients who have cancer [4, 5]. However, study limitations (small sample size, lack of adequate control groups) and conflicting results made firm conclusions impossible [6, 7]. Moreover, while results of earlier studies are encouraging, these studies have not compared the physiologic responses to these treatments and have typically excluded older cancer survivors. Thus, in order to advance this area of research, the next step is to test the feasibility and compare the efficacy of these interventions using physiological and behavioral measures of distress. This pilot study served as a first step to evaluating the use of a Swedish Massage and a Reflexology protocol for relief of distress in nursing home cancer survivors. 

## 2. Materials and Methods

An experimental, repeated-measures, crossover design study of 18 older cancer survivors residing in nursing homes was conducted from 2009–2011. This design was selected because it offered advantages over parallel group trials including: (a) that each subject acted has his or her own control, eliminating among-subject variation; (b) that fewer subjects were required to obtain the same power; (c) that every subject received both conditions [8].

Directors of nursing at 3 large nursing home facilities in Pennsylvania approached residents for permission to be contacted for the study. The medical director at each facility gave final approval to contact the residents, and their responsible party for consent. Subjects were included if they were (a) residents of the nursing home for at least 6 months, (b) aged 75 or over, (c) diagnosed with a solid tumor (lung, prostate, colorectal, breast) in the last 5 years (d) completed cancer treatments, and (d) capable of giving informed consent, or had an acceptable surrogate capable of giving consent on the subjects behalf. Exclusion criteria were based on the relevant literature that outlines suitability for elders receiving massage-based treatments and included [9, 10] (a) evidence of rapid terminal decline, recent traumatic injury, or hospitalization within the 2 weeks, (b) skin diseases: acute psoriasis, eczema, severe bruises, skin infection or ulceration, open wound, recent burn or fracture, (c) inflammatory conditions: acute rheumatoid arthritis, systemic lupus erythematosus, ankylosing spondylitis, Reiter's syndrome, (d) cardiovascular conditions: history of deep vein thrombosis, phlebitis, angina, a pacemaker, (e) recent discontinuation (less than 2 weeks) of physiotherapy that included massage therapy, (f) fever (recent temperature >102° within past 24 hours), or (g) currently prescribed anticoagulant medication (e.g., Coumadin, Heparin, or derivative substances).

Consenting subjects were randomized into two conditions. Those assigned to the first group received one week of friendly visits (for baseline assessment) followed by four weekly sessions of Swedish Massage, a one-week washout period, then 4 weekly sessions of Reflexology. Those assigned to the second group received one week of friendly visits followed by four weekly sessions of Reflexology, a one-week washout, then 4 weekly sessions of Swedish Massage. The protocols were developed based on Standards of practice and expert guidelines of the American Massage Therapy Association (AMTA) and National Certification Board for Therapeutic Massage and Bodywork standards of practice [11]. To reduce the extraneous effects of multiple interventionists, the friendly visits, massage, and reflexology were provided by a single, certified, reflexology/massage therapy provider. The protocols were offered at the same time (between 2 and 4 pm) on the same day each week. All subjects received an equal number and duration of sessions in the privacy of their room in the nursing home. There was no script for any of the sessions. It was normal for the practitioner to converse with the subject and give a brief overview of the session. The subject would normally “lead” the conversation.

### 2.1. Intervention Protocols

The Swedish Massage protocol was prespecified and involved a combination of 10 minutes of light stroking and light pressure using the whole hand to plantar and dorsal surfaces and all tissue from the toes to the knee of each leg (20 minutes total). The Reflexology Intervention was based on the original Ingram method and used a combination of finger pivot and thumb walking techniques to the base of the foot and the toes that correspond with reflex points. The sole, instep, and lateral aspects of the foot were stimulated 5 times, each foot for a total of 10 minutes per foot (20 minutes total). The interventionist was licensed and certified in both modalities and underwent additional training in the detailed protocols and was assessed for fidelity as random intervals [12].

### 2.2. Study Outcomes

Baseline data collection began in September 2009 and included an intake assessment of demographic and medical information. Data collectors blind to group condition performed follow-up data collection. Each data collection encounter was designed to take less than 15 minutes and was completed at four intervals across the day: (1) early morning: 7–7:30 am, (2) mid morning: 11–11:30 am, (3) early afternoon 1–1:30 pm, (4) late afternoon: 4–4:30 pm. These times were selected to maximize the opportunities to observe the subjects mood and to capture the diurnal variation in salivary cortisol, while avoiding interruption of the interventionists' presence.

Over the course of the intervention day, 3 distinct types of data were collected: (1) saliva samples from which salivary cortisol was measured; (2) 5-minute observation of affect (e.g., positive and negative mood) using the Apparent Affect Rating Scale (AARS) [13, 14]; (3) pain using the checklist of nonverbal pain indicators (CNPI) [15, 16]. Measures were then averaged to provide daily mean values for each outcome of interest.

#### 2.2.1. Salivary Cortisol

The primary outcome of interest was physiologic distress as measured daily average salivary cortisol [17]. Since cortisol possesses diurnal qualities, samples (of .5–1 mL volume each) were collected across the day to capture the circadian patterns. Assays were collected using oral swabs made of a nontoxic, inert polymer shaped into a 30 × 10 mm cylinder designed to help filter mucus and other matter from the sample. The swabs were held under the tongue for one minute starting on awakening (7–7:30 am) and at 3 additional intervals (midmorning, early afternoon, late afternoon). Care was taken when collecting saliva to avoid collection after mouth cleaning, meals, snacks, or medications. Saliva samples were transferred to 2 mL cryovials and stored frozen (at least −20°C) until assayed. All samples were assayed for cortisol using a highly sensitive enzyme immunoassay 510 K cleared for use as an in vitro diagnostic measures of adrenal function (Salimetrics, PA). The test had lower limit of sensitivity of .007 ug/dL, range of sensitivity from .007–3.0 ug/dL, and an average intra- and interassay coefficients of variation of less than 5.0% and 10.0%.

**Observation of Affect**

Positive and negative mood was measured by the AARS scale which consists of five items (positive mood: alertness, pleasure; negative mood: sadness, anxiety, anger), requires 5 minutes of observation, and provides reliable and valid readings of positive and negative affect and levels of alertness for both cognitively intact and impaired nursing home residents [13, 14]. Psychometric properties have been well demonstrated in the sample population and documented in earlier studies, including interobserver reliability (ICC = 0.91 for the current study), convergent and discriminant validity, and support for its two-factor (positive mood, negative mood) structure [14].

**Pain**

The CNPI, a behavioral observation scale for nonverbal older adults with cognitive impairment, is one of the more rigorously tested pain assessment instruments [15, 16]. The CNPI is composed of six items (nonverbal vocal complaints, facial grimacing/wincing, bracing, restlessness, rubbing, and verbal vocal complaints), that are rated as presence or absence of pain and has good face validity with verbal, horizontal visual, vertical visual, and faces pain scales, and established interrater reliability for periods of rest and movement [15, 16].

#### 2.2.2. Statistical Analysis

 The feasibility of the study was assessed by examining the rates of recruitment, retention and suitability of the outcome measures. The efficacy of Swedish Massage Therapy versus Reflexology were compared to baseline values for each outcome variable based on subjects who had available data for both pre- and post-treatment time points. The treatment effect size was computed on all change scores with a Cohen's  *d*  [18],based on difference between the averages of the post-treatment values minus the average of the baseline values for each condition. Paired  *t*-tests were used to compare Swedish Massage Therapy versus Reflexology with respect to their mean change baseline to post-treatment values as described above (Table 1). Since cortisol is not normally distributed, all analyses used the log-transformed hormone values; however, nontransformed data are reported in the tables and text to facilitate interpretation.

## 3. Results and Conclusion

Of the 45 residents approached for consent, 20 consented, 12 declined, 11 did not return consents, 1 resident died, and 1 resident was hospitalized while invitations to participate were in the mail. Of the 20 that consented, 2 were hospitalized prior to baseline data collection and were unable to participate, thus we randomly assigned 18 individuals to the 2 groups. The ages of participants averaged 90 years and ranged from 85 years to 98 years. Approximately 66% (*N* = 12) were female and 33% (*N* = 6) were male. Mental status as measured by the Mini-Mental State score ranged from 0 to 18 with an average score of 10.17, indicating significant cognitive impairment. The types of cancer represented in the sample were: breast cancer (*n* = 7,39%), prostate cancer (*n* = 5,28%), colorectal cancer (*n* = 5,28%), and lung cancer (*n* = 1,5%). All study procedures were well tolerated by the study participants. No drop outs occurred during the study period and no adverse events or outcomes were observed.

Table 1 shows the group means and difference scores associated with the outcomes of interest for the Reflexology and Swedish Massage conditions, along with Cohen's  *d*  effect size estimates. Within group, comparison of the treatment results revealed that both conditions were associated with a statistically significant changes in salivary cortisol, negative affect, positive affect, and pain (*P* < .05), when post-treatment values were compared to the baseline values, with a slight advantage indicated for Reflexology. Cohen's  *d*  effect size estimates ranged from *d* = .1 to *d* = .77, and on average were in the medium range of effect [18]. According to between-group  *t*-tests, no significantly greater improvement in outcomes resulted when the two treatment conditions were compared.

## 4. Discussion

The results demonstrated the feasibility of providing and studying manual CAM modalities in nursing home residents with cancer and indicate the need for larger trials. Although our sample size was small, the data suggested some efficacy of the Massage and Reflexology intervention, particularly related to a reduction in observed pain, observed affect, and measures of stress. Although most of the effectsizes noted were in the medium range, a larger study would be needed to determine whether the effect sizes suggested in this pilot study can be confirmed with statistically significant results. Reflexology appeared to offer a slight benefit on outcomes when compared to Swedish Massage, although this benefit was not significant. The mechanism underlying this potential benefit may be attributed to the stimulation of the acupressure points during Reflexology treatments. This hypothetical mechanism may deserve further investigation in a larger trial.

The study has several important limitations. Participants may not be representative of nursing home residents with cancer. The three recruitment sites served a relatively homogenous population of Caucasian older adults. Also, by design the study included only English-speaking residents who were medically stable and not actively undergoing cancer treatments. Thus the subjects may systematically differ from residents with cancer who did not meet eligibility criteria. Moreover, because the sample was not randomly selected from the nursing home population, it is unclear whether the responses we observed are generalizable to other groups of older cancer survivors. In addition, we were unable to assess clinically significant improvements in cortisol, pain, or mood since clinically significant change of the measures used in this study has not been determined. Moreover, the lack of normative data on measures of salivary cortisol in this population limited our ability to test for clinical meaningful changes in this biomeasure. The sample size did not permit us to explore the interaction of covariates in this sample. We were unable to include a usual care control group due to sample size and budget restrictions. Another limitation of this study is the concern that small samples may not be truly representative of the range of subject heterogeneity that can be observed in a larger sample; thus, the effect-size estimates may be larger than would be observed in a larger replication study [19].

These limitations notwithstanding, this pilot study had a number of strengths, which included a randomized design, stringent manualized conditions, and evaluations that were blind to treatment assignment. The results suggest that both Swedish Massage and Reflexology were well tolerated and potentially beneficial in reducing distress and pain and improving mood in older cancer survivors residing in nursing homes. Previous research has supported the value of CAM modalities such as massage and reflexology for relieving distress in older adult patients with cancer and offer guidelines for therapists [20, 21]. For example, the REST study demonstrated significant benefits of massage on pain and mood in adults with advanced cancer [22]. However, given that few clinical trials of massage or reflexology in a frail, institutionalized, older patient population have been published, few direct comparisons are available. Nonetheless, our results confirm earlier studies on CAM modalities in cancer survivors and extend the findings to a sample of participants typically excluded from earlier trials.

These preliminary findings support further study of manual CAM modalities as part of a palliative approach to institutionalized cancer survivors. Developing additional insights into physiological effects and mechanisms of manual CAM interventions is a crucial component of the scientific evidence base needed to guide future clinical practice for older cancer survivors.

## Figures and Tables

**Table 1:** Group means and SDS for outcomes, difference scores (change in treatment values), and effect size estimates.

Outcome of interest	Arm	Means ± SD			ES	*P* ^ °^
		Baseline	Post-treatment	Change from baseline		
Salivary cortisol (ug/dL)^1^		.257 ± 1.1				
	R		.157 ± .09	−0.10^∗^	−.13	
	M		.209 ± .08	−0.05^∗^	−.10	0.23

Positive affect^2^		1.58 ± 0.93				
	R		2.25 ± 0.9	+0.67^∗^	+.73	
	M		1.94 ± 1.0	+0.36^∗^	+.30	0.16

Negative affect^3^		1.17 ± .95				
	R		.823 ± .72	−0.35^∗^	−.42	
	M		.941 ± .82	−0.23^∗^	−.30	0.16

Pain^4^		2.29 ± 1.2				
	R		2.00 ± .79	−0.29^∗^	−.35	
	M		1.58 ± 1.2	−0.71^∗^	−.77	0.22

^1^Higher score: higher physiologic stress.

^2^Higher score: higher positive affect.

^³^Higher score: worse negative affect.

^4^Higher score: higher observed pain

R: Reflexology, M: Swedish Massage; SD: standard deviation; ES: standardized effect sizes.

°*t*-test comparing Reflexology and Swedish Massage Conditions.

^∗^Indicates paired *t*-test results demonstrating significant change from baseline (*P* < .05).
